# Potential Utility of Induced Translocation of Engineered Bacteria as a Therapeutic Agent for Mounting a Personalized Neoantigen‐Based Tumor Immune Response

**DOI:** 10.1002/gch2.202100051

**Published:** 2021-12-16

**Authors:** Ginés Luengo‐Gil, Pablo Conesa‐Zamora

**Affiliations:** ^1^ Clinical Analysis and Pathology Department Group of Molecular Pathology and Pharmacogenetics Institute for Biohealth Research from Murcia (IMIB) Hospital Universitario Santa Lucía c/Mezquita sn Cartagena 30202 Spain; ^2^ Pathology and Histology Department Facultad de Ciencias de la Salud UCAM Universidad Católica San Antonio de Murcia Campus de los Jerónimos, s/n, Guadalupe Murcia 30107 Spain

**Keywords:** bacterial translocation, DNA vaccines, engineered bacteria, immunogenicity, immunotherapy, tumor evasion, tumor neoantigens

## Abstract

Today, an unprecedented understanding of the cancer genome, along with major breakthroughs in oncoimmunotherapy, and a resurgence of nucleic acid vaccines against cancer are being achieved. However, in most cases, the immune system response is still insufficient to react against cancer, especially in those tumors showing low mutational burden. One way to counteract tumor escape can be the induction of bacterial translocation, a phenomenon associated with autoimmune diseases which consists of a leakage in the colonic mucosa barrier, causing the access of gut bacteria to sterile body compartments such as blood. Certain commensal or live‐attenuated bacteria can be engineered in such a way as to contain nucleic acids coding for tumor neoantigens previously selected from individual tumor RNAseq data. Hypothetically, these modified bacteria, previously administered orally to a cancer patient, can be translocated by several compounds acting on colonic mucosa, thus releasing neoantigens in a systemic environment in the context of an acute inflammation. Several strategies for selecting neoantigens, suitable bacteria strains, genetic constructs, and translocation inducers to achieve tumor‐specific activations of CD4 and CD8 T‐cells are discussed in this hypothesis.

## Graphical abstract

A new tailored therapeutic approach is presented here consisting of the use of orally administered engineered bacteria containing tumor neoantigens which have been previously identified by sequencing tumor of an individual patient. The efficient systemic antigen presentation to the immune system is triggered by a bacterial translocation caused by inducing a controlled local inflammation in the gut mucosa.

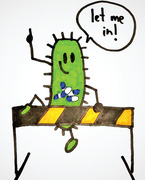

## 1 Perspective

Now that nucleic acid vaccines (NAVs) against SARS‐nCoV‐2 have become the major breakthrough in biomedicine of the XXI century, many researchers and healthcare professionals are wondering whether this knowledge and expertise could be focused on the development of therapeutic vaccines against cancer. Moreover, personalized antitumoral NAVs seem now more feasible than ever, as anti‐COVID NAVs were developed and manufactured in less than one year, which is a reasonable time for the tailored treatment for a given cancer patient. This approach is backed up by recent advances in highly efficacious delivery material for nucleic acids. In this context, one may think that those cancers exhibiting high tumor mutation burden (TMB), such as melanoma and lung cancer, are more eligible for such development. However, even when a tumor expresses a considerable number of neoantigens, these, per se, probably do not make the difference needed to boost an efficient immune response against tumor cells. One successful way to overcome tumor immune evasion has been the use of immune checkpoint inhibitors (ICIs), which increases the level of recognition of neoantigens by the immune system, thus inhibiting anergic signals, such as those driven by PD‐1 and CTLA‐4. Still, the number of patients who could benefit from ICI is limited by the relatively low TMB exhibited by most cancers. Therefore, both NAVs and ICIs encounter the same limitation. If we cannot induce a higher number of tumor neoantigens in a given cancer for obvious reasons, there must be ways to decrease the threshold of immune awareness, thus reaching a similar outcome. One possible answer comes from autoimmune diseases, in which patients show such a low immune threshold that even self‐antigens are attacked by the immune system. Interestingly, CTLA‐4 protein (whose increased activation in certain cancers is repressed by the ICI, ipilimumab) does in fact have decreased activity in certain autoimmune diseases, including inflammatory bowel disease, ^[^ ^1^, ^2^ ^]^ thus suggesting that an autoimmune‐like state could favor the immune response against cancer. Autoimmune diseases and cancer‐associated immune evasion are two sides of the same coin, reflecting the delicate equilibrium between self‐immune reaction and anergy to tumor neoantigens. Two potential questions arise: Could it be possible to induce an autoimmune‐like state in a cancer patient previously inoculated with personalized NAV coding immunogenic neoantigens? Second, could the trigger of this state in fact be the vehicle for NAVs? Evidently, this autoimmune‐like state must be transient, as it must follow the antitumoral effect of acute inflammation and not the protumoral chronic inflammation associated with cancer development. One acute state linked to autoimmune disease that could be mimicked in patients is bacterial translocation, which typically occurs after a local inflammatory reaction in the bowel, thus producing a leakage in the gut mucosa barrier and the access of colonic bacteria to sterile body sites such as blood.^[^ ^3^ ^]^ This phenomenon is highly associated with outbreaks or flares in the context of autoimmune diseases such as psoriasis, inflammatory bowel disease or systemic erythematosus lupus, thus playing causative roles in the immune‐related symptoms of these diseases. Conditions favoring gut barrier loss and bacterial translocation are certain drugs such as antibiotics, chemotherapy agents and irritant substances such as dextran‐sodium sulfate (DSS), which have demonstrated induction of bacterial translocation in non‐autoimmune animal models, including nonhuman primates.^[^ ^4^, ^5^, ^6^, ^7^ ^]^ One of these compounds could be administered to a cancer patient once the transformed bacteria, containing DNA coding from tumor‐specific neoantigens, had found a niche in the colon mucosa. The increased epithelial barrier permeability generated this way would trigger a systemic exposure of bacteria expressing tumor neoantigens to the immune system. Alternatively, engineered bacterial plasmids could be transferred in vivo to host cells (i.e., antigen presenting cells (APCs)) for neoantigen production.^[^ ^8^ ^]^ In any case, this same immune response, exerted by induced therapeutic bacterial translocation (ITBT), would attack tumor cells, creating a peritumoral and intratumoral lymphocytic infiltrate milieu generally associated with better prognosis and survival. Concerning the alteration of the colon mucosa barrier, ITBT can be regulated to cause transient mucosal damage as it has been observed in flares associated with autoimmune diseases such as psoriasis and rheumatoid arthritis.^[^ ^9^, ^10^ ^]^ In these diseases, several approaches proved effective in eliminating “leaky gut syndrome” based on proper diet, supplementation with bioflavonoids, bile acids and the correct administration of antibiotics.^[^ ^11^, ^12^ ^]^ Even the use of DSS in HIV‐infected individuals was found, in two independent studies, to be well tolerated with minor gastrointestinal side effects.^[^ ^13^, ^14^ ^]^ Therefore, intestinal barrier permeability is pharmacologically moldable with many substances used in gastroenterology clinical practice, such as steroids, aminosalicylates, biologics (i.e., anti‐TNF‐α), probiotics, and mucosal protectors, allowing this process to be controllable and reversible.^[^ ^15^ ^]^

**Figure 1:**
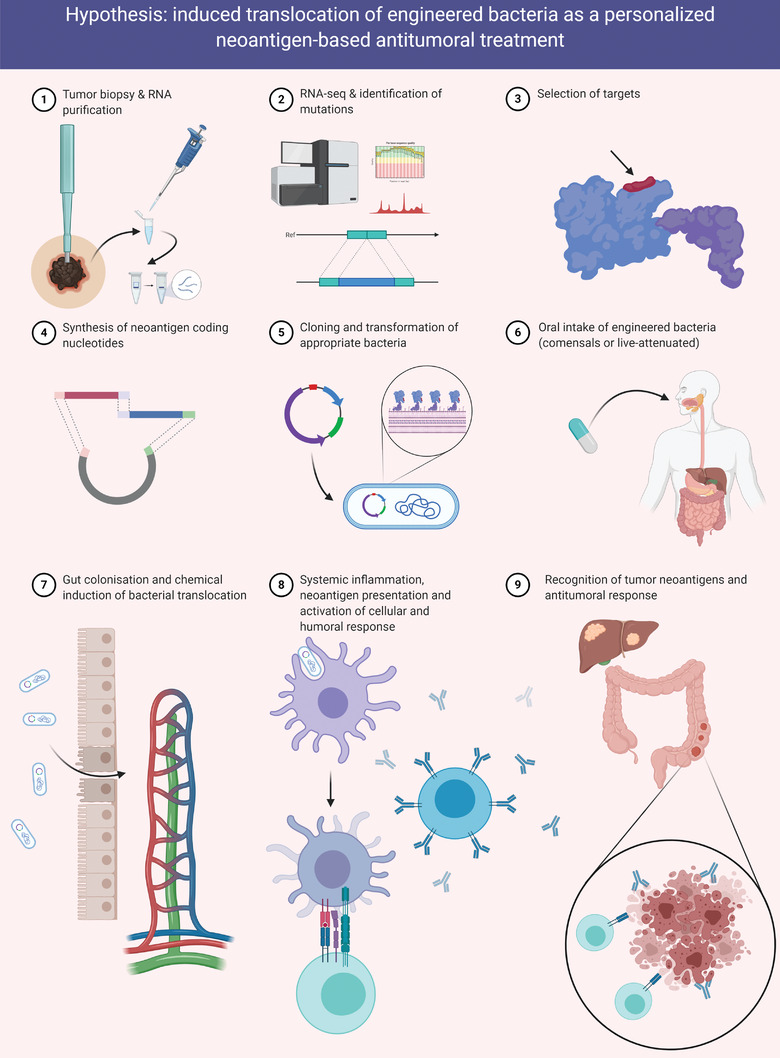
Schematic representation of the procedure based on the hypothesis. Briefly, tumor RNA sequencing renders a number of neoantigens whose coding sequences can be synthesized and cloned on bacteria plasmids. Human gut commensal or live‐attenuated bacteria are transformed with these plasmids and orally administered to the cancer patient. Once bacteria have found a niche on the colonal mucosa, chemical compounds inducing bacterial translocation are administered. This induction will allow bacteria to access blood vessels, lymph nodes and other immune organs, thus eliciting, once neoantigens are presented by antigen‐presenting cells, a CD4 and CD8 T‐cell response against the neoantigen‐expressing tumor cells. Created with BioRender.com

From a practical perspective the design and development of ITBT is a complex process comprising several steps (**Figure** **1**). The first step in selecting candidate neoantigens would be the isolation and massive sequencing of nucleic acids from patient tumor tissue. It could be argued that RNAseq would be preferred to whole genome/exome sequencing (WGS/WES) because it surveys the entire transcriptome including peptides arising from RNA editing. In parallel, RNAseq or WGS/WES would be carried out on normal tissue to discard RNA alterations that were not restricted to the tumor. Several approaches to selecting neoantigens, which will depend on the number of tumor‐associated mutations found, have been elegantly reviewed elsewhere.^[^ ^16^ ^]^ Given that tumors with high TMB usually have abundant TIL and good response to ICI (e.g., melanoma and lung adeno‐ and squamous cell carcinomas), ITBT would hypothetically be more appropriate for treating tumors not characterized by high TMB, such as ovarian, breast, prostate and colorectal cancers, to cite the most common ones. With this proviso, candidate neoantigens identified by RNAseq for ITBT could be left unfiltered to perform unbiased testing of all of them. However, when considering neoantigen production by bacteria, in silico peptide algorithms might be needed to select those neoantigens not potentially subjected to post‐translational modifications such as glycosylation.

Once the neoantigens have been defined, immunological screening assays would have to be performed to ensure that these antigens would be processed and presented by APC, and subsequently recognized by T‐cells.

The second step would involve construct generation and transformation in the appropriate bacteria, which would be administered orally. Intravenous (IV) administration could be another way for engineered bacteria to get access to peripheral blood and several clinical trials (NCT01266460 and NCT02002182: accessed in August 2021 at www.clinicaltrials.gov) have explored this way of administration. However, IV immunization with attenuated bacteria is a more invasive method, and is not exempt from risks associated with the introduction of live microorganisms directly into the circulatory system (which is not a common means of infection). Despite this possibility, no significant problems have been reported related to infections in these trials. One of the concerns associated with the IV setting is the acquired immunity against the cargo microorganism if successive doses are needed. By contrast, oral immunization simplifies the drug administration setting without the requirement of intrahospital IV treatment, since it can be administered in the same health center, or even at home. While oral DNA vaccination mediated by live attenuated bacteria for cancer immunotherapy is not a new concept in oncology, this approach suffers from a low infection efficiency due to endogenous biological barriers that limits the infection process.^[^ ^17^ ^]^ In contrast, the combination of ITBT with DNA oral bacteria vaccination benefits from the interplay between gut microbiota and the immune system which takes place on both sides of the intestinal barrier, and this communication is highly conserved in the evolution. Bacterial translocation through the gut is an interesting and natural entry way of getting genetic information into systemic circulation.

Plasmids containing polycistronic DNA or tandem minigenes (TMG) coding an average of 10 different neoantigens (33–60 base pairs in length each) would be generated and electroporated in bacteria species that are part of the colon microbiota, which are prone to bacterial translocation and naturally have tropism for APCs.^[^ ^18^ ^]^ In fact, previous published data suggest that manipulating the microbiota may modulate cancer immunotherapy.^[^ ^19^ ^]^ The release of the neoantigens in a systemic environment could be triggered by translocated bacteria through different means. One of them, termed bactofection, is the delivery of a plasmid to be transferred to the host cell for production of heterologous antigen, or the production of the heterologous antigen (often followed by secretion) by the bacterial vector itself.^[^ ^18^, ^20^ ^]^ The other one is by the expression of accessible neoantigens, which can be achieved by various strategies, e.g., the genes coding neoantigens can be constructed to be overexpressed on the surface of the bacteria, or even secreted in extremely high concentrations, to attempt recognition of them by the immune system. In this line, Kang and Curtiss in 2003^[^ ^21^ ^]^ demonstrated that in orally immunized mice, the antigen‐specific humoral response of an attenuated *Salmonella typhimurium* carrying PspA derived from the *Streptococcus pneumonia* surface protein was enhanced 10 000‐fold by incorporating the secretion signal from β‐lactamase compared with the unfused PspA construct. Heterologous genes can be fused into gene‐encoding bacterial outer membrane proteins such as OmpA, LamB, or flagellin.^[^ ^22^, ^23^, ^24^ ^]^

Good bacteria candidates could be those belonging to lactobacilli or enterococci genera, such as *Enterococcus gallinarum*, a pathobiont which induces auto‐immunity and colonizes mesenteric veins and lymph nodes, the liver and the spleen in mice and humans.^[^ ^4^ ^]^ Moreover, a recent study showed the antitumor properties of an *E. gallinarum* strain,^[^ ^25^ ^]^ this bacteria being amongst the most enriched species in the stool of patients responding to ICI therapy.^[^ ^26^ ^]^ Attenuated bacterial vectors such as *S. typhimurium* or *Listeria monocytogenes* are good candidates as well, since there are strains engineered to retain plasmids, express heterologous antigens upon inducible factors and display neoantigens on the surface. In addition, *L. monocytogenes* can escape phagosomal degradation via its virulence factors, resulting in an intracellular lifecycle that enables neoantigens to be presented both by HLA‐I and HLA‐II. In addition, these bacteria have shown tropism for tissue conditions associated with cancer growth, such as hypoxic and necrotic microenvironments and “leaky” vessels associated with tumor angiogenesis.^[^ ^18^ ^]^ Various evidences supports that attenuated *L. monocytogenes* is an efficient vector at presenting antigens.^[^ ^27^, ^28^, ^29^, ^30^, ^31^, ^32^, ^33^, ^34^, ^35^, ^36^, ^37^, ^38^, ^39^, ^40^ ^]^

Other conditions needed to be taken into account to favor bacterial translocation are the administration of antibiotics (a 2‐day course of ceftriaxone enables *E. faecalis* and *Lactobacillus* spp. to disseminate to the liver, spleen, and lymph nodes within 3–4 days of exposure, with subsequent clearance after 14 days);^[^ ^41^ ^]^ TLR7 agonists such as imiquimod (experiments in mice demonstrated the importance of stimulating TLR7 signaling to cause *Lactobacillus reuteri* translocation);^[^ ^42^ ^]^ proton pump inhibitors; and chemotherapy agents such as cyclophosphamide or nonsteroidal anti‐inflammatory drugs such as indomethacin.^[^ ^4^ ^]^

Apart from the particular ability of *L. monocytogenes* to stimulate CD8 T‐cells via HLA‐I, translocated bacteria expressing different tumor neoantigens of 11–20 amino acids in length each are able to be presented by HLA‐II molecules on APCs and be recognized by CD4+ T‐cells. These CD4+ responses have proved to be efficient in antitumoral treatments. In fact, in patients with melanoma or glioblastoma, a good response against immunizing peptides favoring CD4+ over CD8+ has been observed, even though these were predicted and prioritized using HLA‐I binding algorithms.^[^ ^43^ ^]^ The use of antigen presentation via HLA‐II is supported by the facts that the majority of the immunogenic mutanome is recognized by CD4+ T cells and that vaccination with such CD4 T cell‐reactive mutations confers strong antitumor activity.^[^ ^44^ ^]^

It is important to stress that ITBT is not exempt from risks associated with systemic inflammatory reactions such as cytokine storms, but neither is the use of conventional chemotherapy, which seriously affects proliferating healthy cells. Furthermore, and in contrast to ITBT, chemoradiotherapy does not provide a specific effect against an individual tumor. At this point is worth mentioning that cytokine storm is generally associated with targeted cellular immunotherapy against hematological malignancies, while is less common following immunotherapy in the treatment of solid tumors. Moreover, the induction of cytokine storm by attenuated bacteria itself is a very rare event. In fact, the aforementioned clinical trials (NCT01266460 and NCT02002182) reported cytokine storm in 12% and 0% patients, probably associated with the antitumoral effect rather than caused by the bacteria infection. On the other hand, ITBT can show reduced effectiveness due to plasmid loss, poor immunogenic neoantigens included in the vaccine and occurrence of additional tumor mutations while the vaccine is being developed. Undoubtedly, the application of ITBT would require fine tuning to obtain a maximal immunogenicity against neoantigens with minimal side effects, including the unlikely conversion of attenuated bacteria into a pathogenic state, which could be solved with specific antibiotics.^[^ ^45^, ^46^ ^]^ This hypothesis could be tested in animal models using, for instance, syngeneic mice and their corresponding established tumor cell lines, in which neoantigens had been identified prior to construction of the expression plasmid. The evaluation of adequate immunization after ITBT could be tested by measuring the production of specific antibodies against neoantigens and analyzing T cell populations. The efficacy of this setup could be tested by implanting subcutaneous or orthotopic models with the same syngeneic cell line and then applying this immunotherapy. The endpoints could be evaluating tumor regression by measuring tumor size and peri‐ or intratumoral lymphocyte infiltration. The comparison would be established with a control group consisting of mice receiving the same treatment, but being administered bacteria with non‐neoantigen‐coding‐plasmid.

As the combination of ITBT and antigenicity prediction tools has never been tested, the limits, in terms of how low TMB could be for this strategy to be effective, are still to be determined. In any case, it represents a hypothesis for treating tumors with such a low TMB, which would pass unnoticed to the immune system with conventional therapy.

All microorganisms that could potentially be used in this therapeutic setting must be sensitive to many antibiotics to prevent some undesired side effects of this treatment. This is easy to achieve in the laboratory, both by choosing naturally sensitive microorganisms or by generating sensitivity in vitro. In conclusion, this hypothesis, based on the Trojan horse concept, merits consideration, and should be explored initially in preclinical studies as a possible way to increase the immune awareness against cancer, especially in those tumors with lower TMB and TILs.

## Conflict of Interest

The authors declare no conflict of interest.

## Author Contributions

P.C.‐Z. had the hypothesis idea, developed the hypothesis, and wrote the draft of the manuscript. G.L.‐G. co‐participated in the development of the hypothesis and in preparing the figure. Both authors revised the current version of the manuscript.
